# Transcriptomic Analysis in Strawberry Fruits Reveals Active Auxin Biosynthesis and Signaling in the Ripe Receptacle

**DOI:** 10.3389/fpls.2017.00889

**Published:** 2017-05-29

**Authors:** Elizabeth Estrada-Johnson, Fabiana Csukasi, Carmen M. Pizarro, José G. Vallarino, Yulia Kiryakova, Amalia Vioque, Javier Brumos, Nieves Medina-Escobar, Miguel A. Botella, José M. Alonso, Alisdair R. Fernie, José F. Sánchez-Sevilla, Sonia Osorio, Victoriano Valpuesta

**Affiliations:** ^1^Departamento de Biología Molecular y Bioquímica, Instituto de Hortofruticultura Subtropical y Mediterranea, Universidad de Málaga-Consejo Superior de Investigaciones CientíficasMálaga, Spain; ^2^Dipartimento di Scienze, Università degli Studi della BasilicataPotenza, Italy; ^3^Department of Plant and Microbial Biology, North Carolina State University, RaleighNC, United States; ^4^Max Planck Institute of Molecular Plant PhysiologyPostdam-Golm, Germany; ^5^Instituto Andaluz de Investigación y Formación Agraria y Pesquera, IFAPA-Centro de ChurrianaMálaga, Spain

**Keywords:** auxin, fruit, strawberry, transcriptome regulation, ripening

## Abstract

The role of auxin in ripening strawberry (*Fragaria* ×*ananassa*) fruits has been restricted to the early stages of development where the growth of the receptacle is dependent on the delivery of auxin from the achenes. At later stages, during enlargement of the receptacle, other hormones have been demonstrated to participate to different degrees, from the general involvement of gibberellins and abscisic acid to the more specific of ethylene. Here we report the involvement of auxin at the late stages of receptacle ripening. The auxin content of the receptacle remains constant during ripening. Analysis of the transcriptome of ripening strawberry fruit revealed the changing expression pattern of the genes of auxin synthesis, perception, signaling and transport along with achene and receptacle development from the green to red stage. Specific members of the corresponding gene families show active transcription in the ripe receptacle. For the synthesis of auxin, two genes encoding tryptophan aminotransferases, *FaTAA1* and *FaTAR2*, were expressed in the red receptacle, with *FaTAR2* expression peaking at this stage. Transient silencing of this gene in ripening receptacle was accompanied by a diminished responsiveness to auxin. The auxin activity in the ripening receptacle is supported by the *DR5*-directed expression of a *GUS* reporter gene in the ripening receptacle of *DR5-GUS* transgenic strawberry plants. Clustering by co-expression of members of the *FaAux/IAA* and *FaARF* families identified five members whose transcriptional activity was increased with the onset of receptacle ripening. Among these, *FaAux/IAA11* and *FaARF6a* appeared, by their expression level and fold-change, as the most likely candidates for their involvement in the auxin activity in the ripening receptacle. The association of the corresponding *ARF6* gene in Arabidopsis to cell elongation constitutes a suggestive hypothesis for *FaARF6a* involvement in the same cellular process in the growing and ripening receptacle.

## Introduction

Fruit ripening is a complex and coordinated irreversible developmental process that leads to the production of a soft and edible ripe fruit. The hormone auxin is known to play a critical role in fruit growth, from flower formation to fruit ripening (Pattison et al., 2014). At early stage is believed to participate in the cell expansion associated to fruit growth, while at maturation a role has also been proposed (Trainotti et al., 2007; McAtee et al., 2013), although not definitive information exits on the cellular and molecular processes involved. Strawberry has been considered as an model to study the role played by the hormone auxin, at the molecular level, in fruit growth and ripening. In strawberry fruit, it was reported that growth of the receptacle ceased if achenes, the source of auxin, were removed after pollination, but growth of the receptacle was taken up again after the external application of 2-naphtoxyacetic acid (Nitsch, 1950). Later in development, the removal of the achenes accelerated some processes associated with fruit ripening (Given et al., 1988). Some studies have identified a number of genes that are down-regulated (Harpster et al., 1998; Aharoni et al., 2002) or up-regulated (Castillejo et al., 2004) after the external application of auxin to developing fruits. However, detailed studies on the content, synthesis, and signaling of this hormone in different fruit parts at different developmental stages are lacking.

Auxin sensing and signaling activity is relatively well established in plants (Weijers and Wagner, 2016). In this vein, some years ago, an F-box protein, TIR1, was identified as an auxin receptor (Dharmasiri et al., 2005; Kepinski and Leyser, 2005). The Arabidopsis genome encodes five other F-box proteins, AFB1-AFB5, that also function as auxin receptors (Dharmasiri et al., 2005; Parry et al., 2009). The auxin receptor TIR1 is part of the SCF^TIR1^ ubiquitin ligase complex and binds auxin directly (Dharmasiri et al., 2005; Kepinski and Leyser, 2005). Efficient auxin binding requires the assembly of an auxin co-receptor complex consisting of TIR1 and an Aux/indole-3-acetic acid (IAA) protein (Calderón Villalobos et al., 2012) that then induces poly-ubiquitination of the Aux/IAA protein and its targeting and degradation in the proteasome (Benjamins and Scheres, 2008). Most species produce a high number of Aux/IAA proteins, with some variability in the protein domains as well as in their expression pattern, that might support a variable range of auxin sensitivity within the plant (Dreher et al., 2006; Salehin et al., 2015; Xie et al., 2015; Weijers and Wagner, 2016). An alternative proteasome-independent auxin signaling pathway, located in the endoplasmic reticulum and the cell wall, has been proposed (Sauer and Kleine-Vehn, 2011). Its contribution to the auxin signaling has been reported in some processes such as cell wall loosening and cell expansion (Ljung, 2013).

Transcriptional regulators known as auxin response factors (ARF) are key elements in the transcriptional response of plants to auxin (Weijers and Wagner, 2016). The ARF proteins bind to *cis*-regulatory sequences (*AuxREs*) in the promoter of a set of auxin-dependent genes, controlling their expression and mediating the auxin-dependent growth and developmental processes (Guilfoyle and Hagen, 2007). Most species have a large family of ARF proteins, divided into subfamilies, that are responsible for the diverse roles played by auxin in different cellular processes (Finet et al., 2012). They typically contain a DNA-binding domain (B3) at the N-terminus flanked by a dimerization domain, a medium domain that mediates transcriptional regulation, and a C-terminus domain (PB1) for oligomerization and Aux/IAA heterodimerization (Guilfoyle, 2015; Weijers and Wagner, 2016). Variants of this domain architecture have been found between and within species (Finet et al., 2012), which contributes to the versatility of the transcriptional response to auxin in many different species and circumstances.

Two major pathways for IAA biosynthesis, the tryptophan-dependent and -independent pathways, have been reported for auxin biosynthesis in plants. However, it is accepted that a significant amount of auxin synthesis in plants is predominantly synthesized by the tryptophan-dependent two-step pathway that has as an intermediate indol-3-pyruvic acid (IPyA) (Brumos et al., 2014). The initial conversion of Trp to IPya is catalyzed by a tryptophane aminotransferase enzyme that includes multiple members in several species (TAA1 and TARs in Arabidopsis) (Stepanova et al., 2008). IPyA is then further converted to IAA by the YUCCA proteins, a family of flavin-dependent monooxygenases (Zhao et al., 2001; Stepanova et al., 2011). The *TAA1*, *TAR*, and *YUCCA* genes show specific expression patterns in several species, including the wild strawberry, associated with the involvement of auxin in different developmental processes (Kang et al., 2013; Brumos et al., 2014; Liu et al., 2014). Starting also from Trp a pathway with an intermediate indole-3-acetamide (IAM) has also been proposed to participate in the auxin biosynthesis plants, since both the IAM and the corresponding enzyme activities isolated in Arabidopsis and tobacco (Mano et al., 2010). In general, several mechanisms have been proposed to control auxin homeostasis, including IAA metabolism (biosynthesis, degradation, and conjugation), transport, and compartmentation (Ljung, 2013). Recently, an enzyme controlling IAA oxidation in Arabidopsis has been reported (Porco et al., 2016). In addition, a proportion of cellular auxin is conjugated to other molecules, being proposed that they might play a role of auxin storage molecules, or intermediates compounds in the auxin catabolic pathway (Ljung, 2013). Interestingly, some of these auxin-conjugates have been identified in fruits such as grape and tomato, with values of content changing along ripening (Böttcher et al., 2010).

The complexity of the auxin biosynthesis, sensing and signaling machinery, present in all plant species, makes it obligatory to have global information about their components, their variability, and their occurrence in relation to the process to be studied. RNA-seq analysis allows having this global information, at the transcript level, in a single experiment. A basic requirement for using this approach is the availability of the genomic sequence of the species. However, it has also been reported that mapping the reads in a closely related model species provides valid results (Conesa et al., 2016). Thus, in the case of *Fragaria ananassa*, with no sequenced genome, the sequenced *F. vesca* genome can be used to map the reads and obtain the transcription atlas of a process such as fruit ripening. The *F. vesca* map has been successfully used in the less-related species *Rubus* sp. (García-Seco et al., 2015). Transcriptomic studies in *F. ananassa* are complicated by the fact that it is an octoploid species (Tennessen et al., 2014). This ploidy might significantly increase the number of allelic variants that exist for the different components of the auxin sensing and signaling machinery. A partial solution to this challenge can be provided by the RNA-seq data as some methods have been reported that facilitate transcriptome assembly from RNA-seq data in species without a sequenced genome (Chevreux et al., 2004; Grabherr et al., 2011).

Our study is focused on expanding current knowledge about the role of auxin in strawberry fruit development and ripening. Our RNA-seq analysis of the ripening achene and receptacle identified the red stage-specific expression of selected members of the auxin synthesis, sensing and signaling pathways. This result was accompanied by the measurement of the auxin content in the receptacle at three developmental stages, and a functional test of the machinery by transient silencing of a main auxin synthesis gene. When taken together, our results support the involvement of auxin in the specific cellular processes taking place in the ripening receptacle.

## Materials and Methods

### Plant Material, Plasmid Construction and Transient Transformation

Strawberry plants used for transient transformation were grown and maintained under glasshouse conditions (IFAPA-CIFA Churriana, Málaga, Spain). The transient silencing of *FaTAR2* by agroinfiltration with the RNAi construct pBI-FaTAR2i, or pBI-intron as a control, was carried out in octoploid strawberry (*Fragaria* ×*ananassa* Duch.) cv Camarosa fruits, as described previously (Hoffmann et al., 2006), from March to May.

For the construction of pBI-FaTAR2i, a sequence of *FaTAR2* (KY509034) was PCR-amplified from strawberry (*Fragaria* ×*ananassa*) cv Camarosa cDNA using gene- specific primers (forward, 5′ CTTGACCAACACCACTGAAA-3′; reverse, 5′ GTGTCTTCCTCCTCGGGTCA-3′). The forward and reverse primers contained an Nhel/SpeI and an XbaI/SacI restriction site, respectively. The fragment was ligated into the binary vector pSK that contained XbaI/NheI and SpeI/SacI restriction sites separated by an intron from strawberry (Hoffmann et al., 2006); the vector was thus cut with XbaI and NheI, and the fragment was ligated in the sense direction. Second, the vector was digested with SpeI and SacI, and the fragment was inserted in the antisense direction, obtaining the intron–hairpin construct pSK-TAR2i. This plasmid was cut with AscI, and the digested fragment was cloned into the AscI-cut pBI121, obtaining the construct pBI–FaTAR2i. The fruits remained attached to the plants after agroinfiltration. Seven to 10 days after injection, the fruits were harvested.

The strawberry cv Camarosa fruits used for RNAseq were harvested in four different developmental stages corresponding to green (G), white (W), turning (T), and red (R). These fruits were collected from plants that were grown under field conditions in Huelva, Spain. All fruits were frozen immediately in liquid nitrogen, and achenes were removed using a scalpel on frozen fruits. Transcriptome analysis was performed in three separate pools of 20 fruits each. Each pool was from four different plants. Libraries were sequenced on Illumina HiSeq2000 lanes using 2 × 100 bp reads. More than 30 million reads were generated for each sample. Analyses of transcript data were performed using FastQC, TopHat, Cufflinks, Blast2go, and IGV software as previously described (Trapnell et al., 2012; Sánchez-Sevilla et al., 2014). Normalized RNAseq fragment counts were used to measure the relative abundances of transcripts, expressed as fragments per kilobase of exon per million fragments mapped (FPKM).

Achenes of green strawberry fruits on the plant were carefully removed, using the tip of a scalpel blade. Fruits were harvested at 0, 24, 48, 72, and 96 h after treatment, immediately frozen in liquid nitrogen and stored at -80°C. The injection with NAA was performed in fruits at the turning stage with ca. 250 μl of a basic water solution (NaOH 50 μl in 500 ml of water) 2 mM in naphthalene acetic acid (NAA) and 2% DMSO. Mock solution contained the basic water with DMSO. Fruits were harvested after 24, 72, and 96 h, immediately frozen and stored at -80°C.

### Auxin Determination

Indole-3-acetic acid (IAA) was identified by co-elution with an [^2^H_5_]-IAA standard (OlChemim Ltd, Olomouc, Czech Republic). Recovery experiments in which the amount of authentic IAA added at the start of the experiment was doubled yielded a result of 89.5 ± 2.1%, indicating a high stability of the metabolite and its derivative throughout the extraction, derivatization, and analytical processes.

Indole-3-acetic acid was extracted overnight from 6 g of receptacle fruits without achenes in 20 ml of 80% methanol. After extraction, each sample was reduced in vacuo and diluted with 20 ml of water. The aqueous phase was adjusted to pH 2.8 with 1 M HCl and partitioned four times with equal volumes of ethyl acetate. The ethyl acetate extracts were combined and evaporated to dryness. The residue was dissolved in 1 ml of 10% methanol and applied to a pre-equilibrated C18 cartridge^1^. The column was washed with aqueous acetic acid (pH 3.0), and then IAA was eluted with 80% methanol. After evaporation to dryness, the samples were derivatized and analyzed using internal [^2^H_5_]-IAA standard by GC-MS as in Osorio et al. (2011).

### RNA Extraction, Transcriptome Analysis by RNAseq, and Gene Expression Analysis by Quantitative Real-Time PCR (qRT-PCR)

RNA extraction and transcriptome analysis by RNAseq were performed as previously described by Vallarino et al. (2015). A total of 10 independent samples corresponding to achene and receptacle, at four developmental stages (green, white, turning, and red), leaf and root, with three replications per sample, were analyzed. The total number of reads was over 990 million, and the average of reads per sample ranged from 26,4 to 40,9 millions (Supplementary Table S1). For gene expression analysis by qRT-PCR, first-strand cDNA synthesis of 1 mg of RNA in a final volume of 20 mL was performed using the iScript cDNA synthesis kit (Bio-Rad). Expression of the *FaTAA1, FaTAR2, GUS, FaAux/IAA11*, and *FaTIR1* genes was analyzed by real-time qRT-PCR using the fluorescent intercalating dye SsoFast EvaGreen supermix in the MyiQ detection system (Bio-Rad). Relative quantification of the target expression level was performed using the comparative *C*t method. The following primers were used: for analysis of *FaTAA1* (forward, 5′-GGCCAGTGGATGAGCTATGT-3′; reverse, 5′-CCACCAGGAGAAGTGAGAGC-3′); *FaTAR2* (forward, 5′-TGAGGAACTTGCTTGTGCTG-3′; reverse, 5′-TGGACCT CTCTGCTTCTGGT-3′); *FaAUX/IAA11* (forward, 5′-TGGT GGTCAGGAGCATGATA-3′; reverse, 5′-TTAGCCTCTTCA CGGAACTAAGA-3′); *FaTIR1* (forward, 5′-AGCCACTTG ATGAGCCACTTGATGTGGGTTTC-3′; reverse, 5′-AAAGC GCCTTATCACCAAAA-3′); *FaARF6a* (forward, 5′-AGTTT GTAAATAGTGTGTGGTGCAT-3′; reverse, 5′-CTGCATTGG GACAGACTTCAG-3′); and *GUS* (forward, 5′-GATCGCGA AAACTGTGGAAT-3′; reverse, 5′-AAAGACTTCGCGCT GATACC-3′). To normalize the gene expression levels for differences in the efficiency of cDNA synthesis, transcript levels of the constitutively expressed gene *FaGAPH* (Salvatierra et al., 2010) and/or *FaCHP1* (Clancy et al., 2013) were measured.

### GUS Staining

For GUS analysis, tissues were incubated O/N at 37°C with GUS buffer (50 mM sodium phosphate buffer, pH 7.0, 10 mM Na_2_EDTA, 0.5 mM K_4_ [Fe(CN)_6_]⋅3H_2_O, 0.5 mM K_3_[Fe(CN)_6_], 0.1% Triton X-100, and 1 mg/mL X-Gluc) as previously described (Jefferson et al., 1987) and were then de-stained by incubating in ethanol/acetic acid 3:1 at RT for 24 h.

### FaTAA1 and FaTAR2 Activity Assays

The *FaTAA1* (KY509033) and *FaTAR2* (KY509034) open reading frames were subcloned into pENTR/D-Topo and transferred into pDEST15 by Gateway LR recombination (Invitrogen) as previously described (Stepanova et al., 2008). GST-FaTAA1 was expressed in the BL21 Star (DE3) pLysS strain of *Escherichia coli* (Invitrogen) and induced by 0.5 mM isopropyl b-D-1-thiogalactopyranoside for 4 h. Equal volumes of protein extracts were loaded onto a native 10% polyacrylamide gel and run for 2 h at 100 V at 48°C. The in-gel aminotransferase activity was assayed as described (Pedraza et al., 2004) at 24°C over-night. Biochemical characterization of FaTAA1 and FaTAR2 was performed using recombinant GST-FaTAA1 and GST-TAR2 batch purified on glutathione-sepharose beads (Amersham Pharmacia) according to the manufacturer’s recommendations. Purified protein concentrations were estimated by SDS-PAGE followed by Coomassie Brilliant Blue staining. For a 100-mL reaction, 5 mg of FaTAA1 and FaTAR2 was used. Aminotransferase activity was assayed as previously described (Pedraza et al., 2004).

For HPLC-MS chromatography, reactions were carried out with the purified enzyme at 37°C for 1 h using previously described conditions (Koshiba and Matsuyama, 1993). To identify the products of the enzymatic reaction, indole-3-pyruvic acid and tryptophan (Sigma) were employed as standards. HPLC analysis was performed using a Shimadzu LC-MS 2010 EV module system with a UV photodiode array (190–800 nm) detection. For LC-MS analysis, the same system in combination with a single-stage quadrupole mass analyser coupled with electrospray ionization was utilized. The ion chromatograms were obtained using MS detection with negative ionization. Scans of peaks were stored from m/z of 50 to 2000 amu. The speed of the scan was 2000 amu/second. We used 0.1% acetic acid (HPLC grade) (solvent A) and acetonitrile (GC-MS grade) (solvent B) (VWR) as solvents for HPLC and LC-MS. The gradient program consisted of ratios of solvent A to solvent B as follows: 10:90 (0–5 min), 15:85 (5–10 min), 17:83 (10–20 min), 25:75 (20–30 min), 35:65 (30–40 min), 45:55 (40–50 min), 52:48 (50–55 min), 55:45 (55–58 min), 60:40 (58–58.5 min), 90:10 (58.5–59 min), and 10:90 (59–60 min). Data from LC chromatograms, UV spectrum, ion chromatograms, and MS spectrum were acquired from 0 to 60 min. After each run, the column was washed for 10 min using the solvents with the ratio of solvent A to B of 10:90. A ZORBRX Eclipse XDB-C18 reverse column (4.6 mm × 250 mm, 5-micron) (Agilent) was used to separate compounds from enzymatic reactions. The flow rate was 0.4 ml/min. The injection volume of samples was 30 μl for HLPC assay and 10 μl for LC-MS assay. We used 2 μl standards of freshly prepared IPA and Trp (0.1 μg/μl) to identify the product of the Trp aminotransferase reaction. Reaction products were identified by retention time, UV spectrum, ion chromatograms, and MS spectrum. Each experiment was performed in triplicate.

### Phylogenetic Analysis

The unrooted phylogenetic tree shown in Supplementary Figures S1, S2 online was constructed using MEGA 5.05^2^ with the neighbor-joining statistical method and bootstrap analysis (1000 replicates). Gene sequences were downloaded from Phytozome^3^ and GDR^4^; sequence alignment was performed using Clustal Omega^5^.

## Results

### Expression of Auxin-Synthesizing Genes Supports the Synthesis of This Hormone in Green Achene and Red Receptacle

**FIGURE 1:**
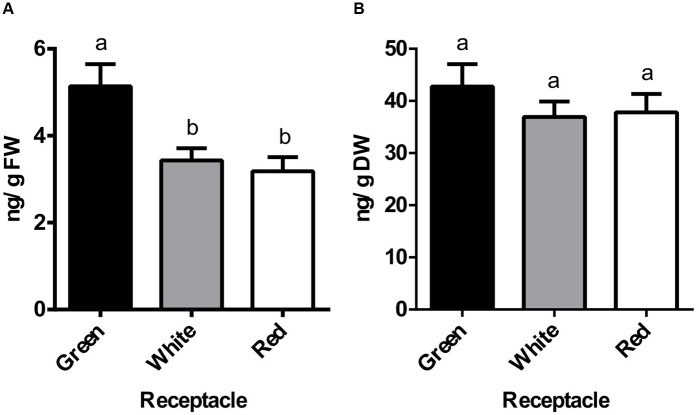
Changes in auxin endogenous levels during receptacle development and ripening. Endogenous levels of indole-3-acetic acid (IAA) in green, white and red receptacle as measured by GC-MS and expressed per gram of fresh weight (FW, **A**) or dry weight (DW, **B**). Bars represent the mean of four independent biological samples ± SE. Different letters indicate a significant difference between samples according to the corresponding ANOVA (*P* < 0.05).

Previous studies of genes expressed in strawberry fruits identified some components of the auxin signaling pathway that were expressed not only in the green fruits but also in the red fruits (Bombarely et al., 2010). As this pattern could be an indication of auxin presence in the ripe receptacle, the content of this hormone was evaluated at three developmental stages. Thus, we measured the content of free IAA in the receptacle at green, white and red stages using GC-MS. The highest IAA content (5.14 ng/gFW) was observed in the green receptacle, and it was then diminished in the white and red receptacles (3.43 and 3.18 ng/gFW, respectively) (**Figure 1A**). This pattern of diminution in the transition from green to white and red stages was altered when the values were expressed on a dry weight basis (**Figure 1B**). In the transition from green to a red receptacle, the total number of cells remains constant with an increase in cell size, mostly by water uptake (Cheng and Breen, 1992). The maintenance of an equivalent amount of free auxin content per dry weight in the transition from green to red receptacle must be the result of either an active transport from the achene or the synthesis of auxin in the receptacle, concurrent with the development of this organ.

In an effort to elucidate the role played by auxin in the ripening of strawberry fruit we performed RNA-seq in achenes and receptacle at different developmental stages (green, white, turning, and red) to identify differential expression of auxin related genes (Supplementary Table S2). Although the strawberry (*Fragaria* × *ananassa*) genome has not been sequenced, the genome of its wild relative *F. vesca* was sequenced (Shulaev et al., 2011), and more recently updated (Tennessen et al., 2014). Mapping the reads of an *F. ananassa* RNA-seq study to the *F. vesca* genome gives valid information about the gene expression of the octoploid species, as previously reported for another less closely related species such as *Rubus* sp. (García-Seco et al., 2015).

**FIGURE 2:**
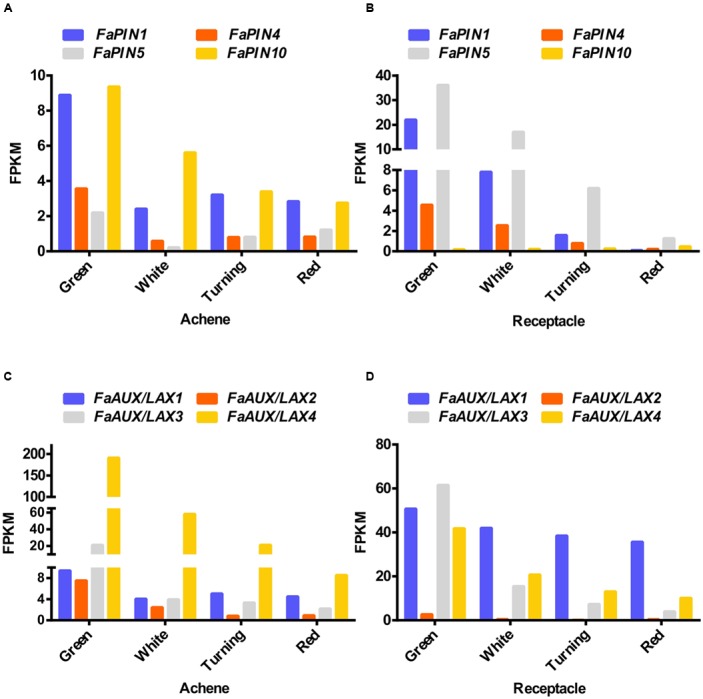
Expression of the four members of the *FaPIN* **(A,B)** and *FaAUX/LAX* **(C,D)** gene families in the achene **(A,C)** and the receptacle **(B,D)** at four developmental stages (green; white; turning; red), by RNAseq. FPKM, fragments per kilobase of exon per million fragments mapped.

Auxin distribution by polar auxin transport is mediated by the PIN and AUX/LAX proteins (Vanneste and Friml, 2009). In the sequenced genome of the wild strawberry *F. vesca*, 10 *FvPIN* (Kang et al., 2013) and four *FvAUX/LAX* (Tennessen et al., 2014) (Supplementary Figure S1) genes have been identified. Only four *FaPIN* genes (*FaPIN1*, *FaPIN4*, *FaPIN5*, and *FaPIN10*) were expressed in the fruits of the cultivated strawberry (*Fragaria* ×*ananassa*), showing a developmental-specific pattern (**Figures 2A,B**). While *FaPIN10* had higher expression in the achene than in the receptacle, the other three *FaPIN* genes displayed higher expression in the receptacle. In all cases, the pattern corresponded to decreased expression in the transition from the green to red stage, both in the achene and the receptacle. Regarding the *F. ananassa AUX/LAX* genes (*FaAUX/LAX1*, *FaAUX/LAX2*, *FaAUX/LAX3*, and *FaAUX/LAX4*), all were expressed in the receptacle and achenes (**Figures 2C,D**). In achenes, the highest expression corresponded to *FaAUX/LAX4*, while the other three members showed higher expression in the receptacle. In all cases, as occurred for *FaPIN* genes, the pattern was a continuous decrease from green to red stages in both the achene and receptacle. It is remarkable that most of the transport genes showed higher expression in the receptacle compared to the achene.

**FIGURE 3:**
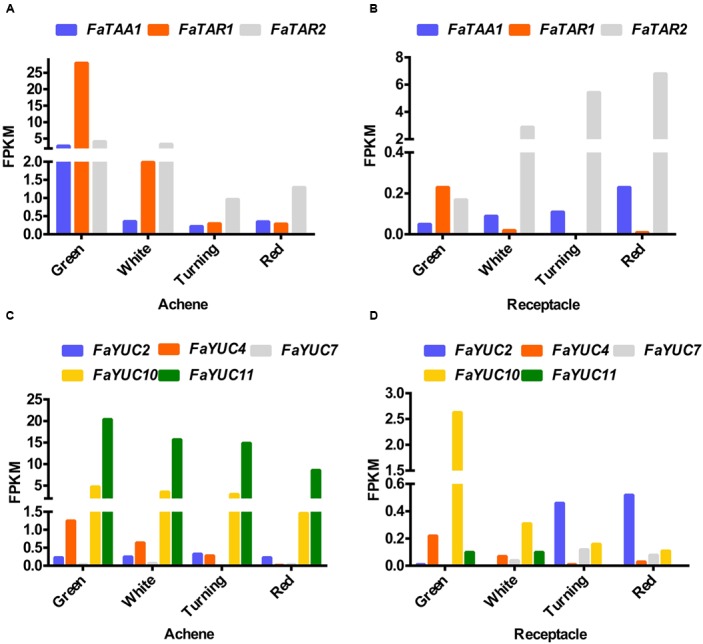
Expression of the three members of the tryptophan aminotransferase gene family (*FaTAA1, FaTAR1*, and *FaTAR2*) and four members of the flavin-dependent monooxygenases (FaYUC2, FaYUC4, FaYUC7, FaYUC10, and FaYUC11) in achene **(A,C)** and receptacle **(B,D)** at four developmental stages (green; white; turning; red), by RNAseq. FPKM, fragments per kilobase of exon per million fragments mapped.

The synthesis of auxin in the Trp-dependent pathway in Arabidopsis involves two steps that are catalysed by (i) the enzyme tryptophan aminotransferase (TAA and TAR), which converts tryptophan to indole-3-pyruvic acid (IPyA), and (ii) a family of flavin-dependent monooxygenases (YUCCA) that convert IPyA to indole-3-acetic acid (Stepanova et al., 2008, 2011). The TAA and YUCCA proteins jointly form a two-step biosynthetic route that constitutes the main auxin biosynthesis pathway in Arabidopsis. In addition, a number of studies in different species suggest that this pathway might be functional in fruits (Pattison et al., 2014). In the *F. vesca* genome, four tryptophan aminotransferases have been mapped (Supplementary Figure S2). The expression of the corresponding *F. ananassa* genes, analyzed by RNA-seq, shows that only three (*FaTAA1, FaTAR1*, and *FaTAR2*) are expressed in fruits (**Figures 3A,B**). In the achene, the three genes show the same pattern, i.e., a steep decrease in the transition from the green to the white stage (**Figure 3A**) and then decreasing to the red stage. The highest absolute value for expression is shown by *FaTAR1*. In the receptacle, the only gene showing expression over 1 FPKM is *FaTAR2* (**Figure 3B**). Interestingly, the expression of this gene continuously increases from the green to the red stage. In relation to the *YUCCA* genes, only five of the nine genes mapped in *F. vesca* (Kang et al., 2013) are expressed in *F. ananassa* (**Figures 3C,D**). In the achene, the common pattern is a decrease from green to red, with the highest expression level being that of the *FaYUC11* gene (**Figure 3C**). Even with low expression, some *YUCCA* genes were identified in the red receptacle (**Figure 3D**).

Synthesis of auxin in plants from Trp has also been reported through the IAM pathway (Mano et al., 2010). A gene encoding a putative indole-3-acetamide hydrolase (AMI1) was identified in the *F. vesca* genome. The expression of the corresponding *F. ananassa* gene showed a decreased pattern from green to red stage in both achene and receptacle (Supplementary Table S2). Its contribution to the auxin content cannot be disregarded, mainly at early developmental stages. In addition to synthesis, homeostasis of auxin is maintained by the hormone conjugation and catabolism. The conjugation of auxin to amino acids is catalyzed by GH3 proteins (Staswick et al., 2005). Six members of the family were expressed in strawberry fruits showing a gene-specific pattern (Supplementary Table S2). In general, their expression is higher in achene. In receptacle, highest expression corresponds to *FaGH3.1*, that dramatically decreases from green to red stage. It is noteworthy than in grape berry the expression of the corresponding *VvGH3.1* increased with ripening (Böttcher et al., 2010). This apparent discrepancy must be analyzed considering that the strawberry *GH3* is a gene family, with gene-specific patterns, and the function of the auxin-conjugates is still under study (Ljung, 2013). There has also been reported the occurrence of amido-hydrolases in Arabidopsis (ILR1, ILL2, and IAR3) that convert back to free auxin some amino acid-conjugated forms of this hormone (LeClere et al., 2002). Two genes with high homology to these amido-hydrolases were identified in the *F. vesca* genome, and the corresponding *F. ananassa* genes showed a tissue- and developmental-specific pattern (Supplementary Table S2). The highest changes along fruit development were found for *FaILR1* with a sharp decrease in expression from green to red achene, while expression in receptacle peaked at the turning stage. Interestingly, the presence of amide-conjugates of auxin in strawberry fruit has been reported long before, and changes in its content varied significantly only in achenes (Archbold and Dennis, 1984). Their function as a source of auxin must be considered, taking account the changes here reported on the expression of putative auxin-conjugating and de-conjugating enzymes.

Regarding auxin catabolism, a *F. vesca* gene with very high homology to the Arabidopsis *AtDAO1* (Porco et al., 2016) was identified. The expression of the corresponding *F. ananassa* gene was higher in achene than in receptacle, increasing with maturation, and minor changes were observed in receptacle (Supplementary Table S2). Its contribution to auxin homeostasis in strawberry requires the confirmation of the enzyme activity and substrate specificity of the gene product.

Altogether, the expression of the auxin-synthesizing genes in strawberry fruits is directed to two organ/stage-specific points of active auxin biosynthesis, the green achene and, apparently at a lower level, the red receptacle. It is accepted that in the green fruit, the auxin synthesized in the achenes provides the hormone for the growth of the receptacle (Nitsch, 1955). The expression patterns of auxin transporters reported here (**Figure 2**) would support an active auxin transport in the receptacle. However, the synthesis of this hormone in red receptacle was unexpected. Therefore, focus was addressed to this organ, characterizing in more depth the synthesis and action of auxin in the ripening receptacle.

We first confirmed by qRT-PCR the expression of *FaTAA1* and *FaTAR2* in the ripening receptacle. The gene-specific primers designed from the assembled transcripts in the RNA-seq analysis (Grabherr et al., 2011) were used. The expression values obtained (Supplementary Figure S3) validated the previously found RNA-seq data (**Figure 3B**). In the transition from the green to the red receptacle, there is a significant increase in the expression of both *FaTAA1* and *FaTAR2*. The increase was higher for *FaTAR2*, which also showed expression levels that were higher than those of *FaTAA1* in the receptacle (**Figure 3B**).

### FaTAA1 and FaTAR2 Have Tryptophan Aminotransferase Activity *In Vitro*

**FIGURE 4:**
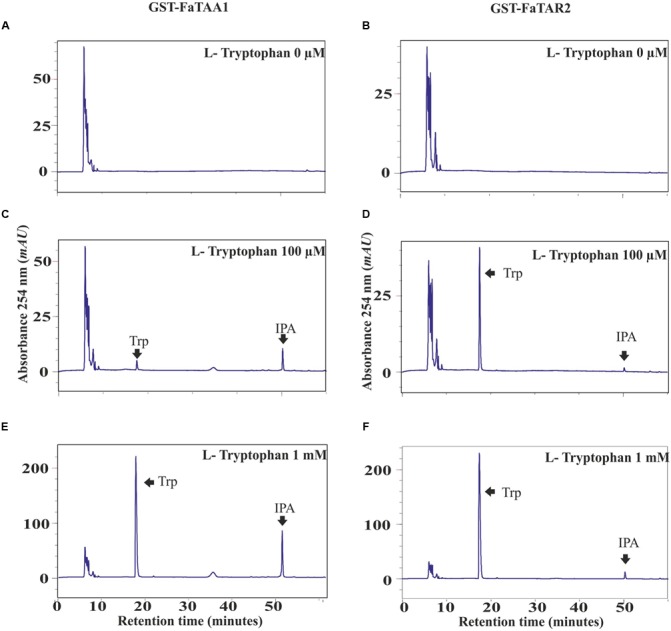
Trp aminotransferase (AT) activity of FaTAA1 and FaTAR2. HPLC chromatograms of the products of the *in vitro* AT reactions catalyzed by the purified GST-FaTAA1 **(A–C)** and GST-FaTAR2 **(D–F)** protein.

The Trp aminotransferase activity of AtTAA1 has been studied in Arabidopsis to confirm its involvement in the production of IPyA from Trp (Stepanova et al., 2008). Thus, full-length cDNAs were cloned for *FaTAA1* and *FaTAR2* to produce the corresponding enzymes in *E. coli*, as described in the “Materials and Methods” section. The GST-purified proteins were assayed for aminotransferase activity in the presence of different concentrations of Trp, as previously described (Stepanova et al., 2008). The reaction products for FaTAA1 were analyzed by HPLC (**Figures 4A,C,E**). In the presence of Trp, two single peaks, corresponding to the enol and keto forms of IPyA, appeared at the end of the incubation period. Their identities were confirmed by HPLC analysis of the external standard. The controls, with boiled enzyme preparation and *E. coli* extract, are reported in Supplementary Figure S4. The transferase activity of the purified protein was also confirmed in gel, using a nitroblue tetrazolium dye (NBT) (Supplementary Figure S5).

The same protocols were followed for the FaTAR2 protein. The chromatograms after incubation of the purified fusion protein GST-FaTAR2 with increasing concentrations of the substrate are shown in **Figures 4B,D,F**. At the highest concentration assayed, 1 mM Trp, a single peak corresponding to the more abundant enol form of IPyA was detected. The controls are shown in Supplementary Figure S4. The Trp aminotransferase activity of FaTAR2 was apparently lower than the activity of FaTAA1. However, this cannot be concluded from the present results as the protein concentration and purification degree of the two GST-fused enzymes, FaTAA1 and FaTAR2, were not evaluated. These results confirm that FaTAA1 and FaTAR2 work as Trp aminotransferases in strawberry as previously described in Arabidopsis, indicating that they are involved in auxin biosynthesis.

### GUS Activity and Expression in Fruits of Transgenic *DR5-GUS* Strawberry Support Auxin Responsiveness in Green and Red Receptacle

**FIGURE 5:**
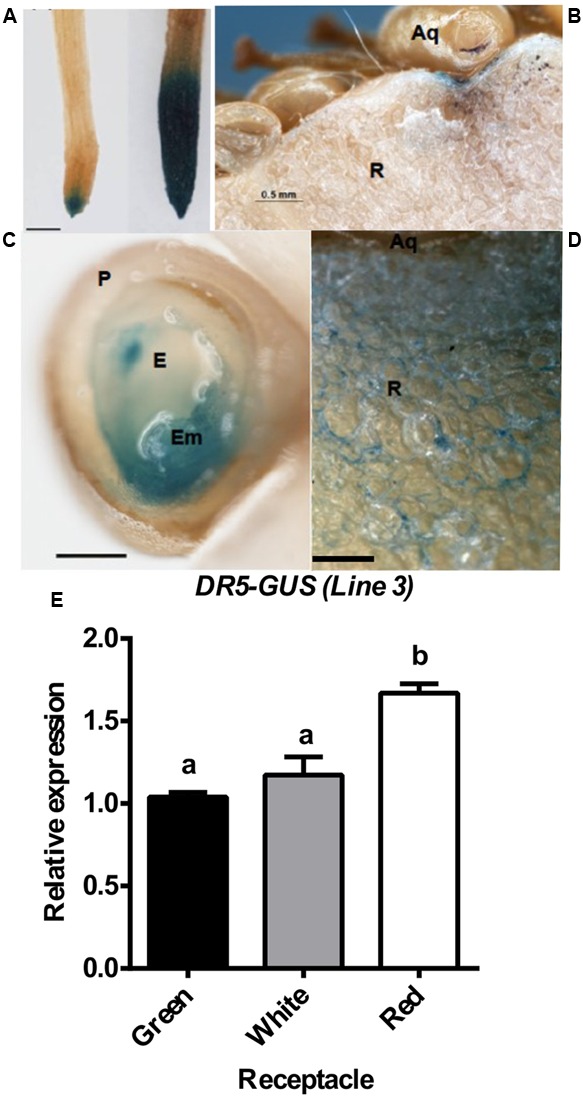
Auxin distribution in strawberry. *DR5-GUS* expression pattern in strawberry roots **(A)** and green fruits **(B–D)**. For the NAA treatment, in **(A)**, plants were grown *in vitro* and treated with a solution of 50 μM NAA in water for 24 h (right), or just with water for control plants (left). Scale bars **(B)** = 500 μm, **(C,D)** = 250 μm,. P, pericarp; E, endosperm; Em, embryo; Aq, achene; R, receptacle. **(E)** Relative expression by qRT-PCR of the *GUS* gene in receptacle at three developmental stages. Different letters indicate a significant difference between samples according to the corresponding ANOVA (*P* < 0.05).

To investigate the role played by auxin in strawberry fruit development, the synthetic auxin response promoter *DR5* (Ulmasov et al., 1997; Ottenschlager et al., 2003), fused to the β-glucuronidase (*GUS*) gene was used to generate transgenic strawberry plants. The roots of the transgenic strawberry plants showed a GUS expression pattern that was similar to that observed in the corresponding transgenic Arabidopsis (Ottenschlager et al., 2003) (**Figure 5A**, left), indicating that GUS expression in DR5-GUS transgenic strawberry plants is a bona fide indicator of auxin output signaling. Moreover, treatment with NAA increased the GUS staining in the root (**Figure 5A**), indicative of a functional response of the DR5 promoter to auxin in this organ. In the green fruit, DR5-driven GUS staining was observed in the receptacle around the achene attachment (**Figure 5B**). A closer view of a sectioned achene showed strong GUS staining in the embryo (**Figure 5C**). In the green receptacle surrounding the achenes, we observed a gradient of GUS staining that initiated at the achene (**Figure 5D**). Several attempts to visualize GUS activity in the red receptacle failed, as we always obtained a dispersed faint blue color that was not clearly visualized (Supplementary Figure S6). Either a dilution effect or the restriction of the activity to a limited set of cells could explain the weak stain obtained in the red receptacle. Then, *GUS* transcription was evaluated in the receptacle at three stages, as this process could overcome these problems. The result showed that in the red receptacle, the expression was maintained, and slightly higher, compared to the green and white stages (**Figure 5E**). Overall, the pattern of GUS activity and *GUS* expression in strawberry fruits confirms the previously reported auxin activity in the achene and green receptacle and the auxin responsiveness in red receptacle.

### Auxin Signaling in the Receptacle Is Mediated by a Stage-Specific Set of *Aux/IAA* and *ARF* Genes

**FIGURE 6:**
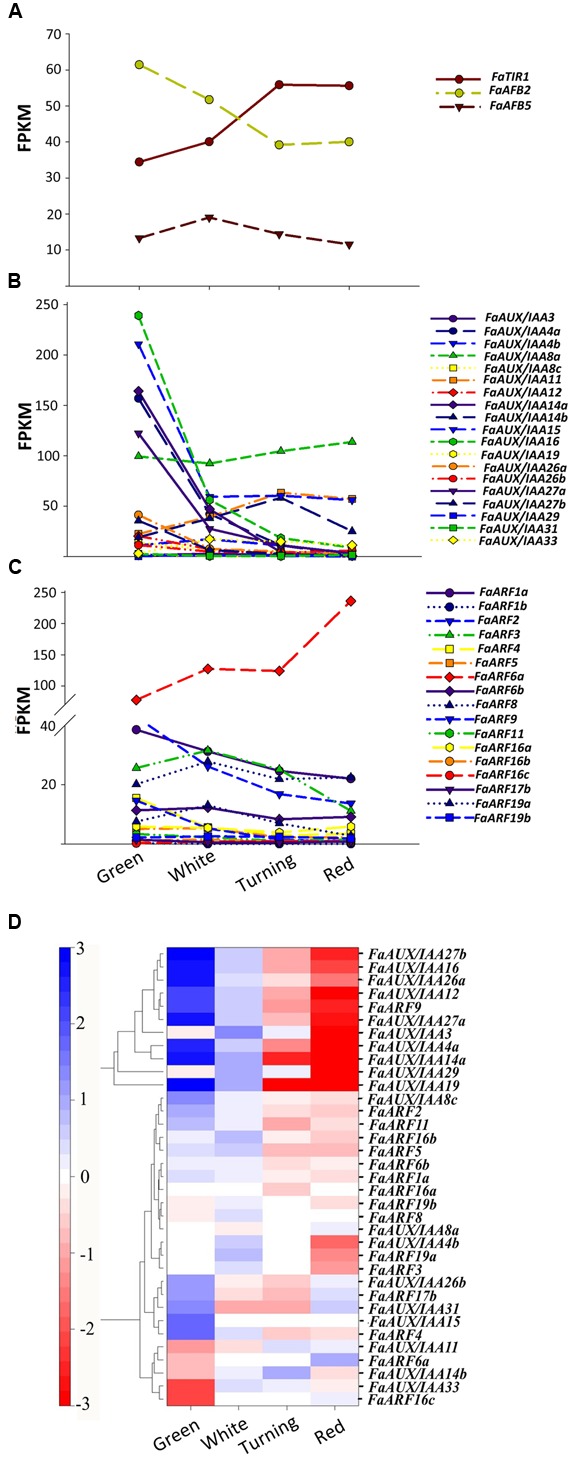
Expression of the gene family of auxin receptors **(A)**, repressors (*FaAUX/IAA*; **B**), and transcription factors (*FaARF*; **C**) in the receptacle at four developmental stages (green; white; turning; red) by RNAseq. FPKM, fragments per kilobase of exon per million fragments mapped. **(D)** *K*-means clusters of 35 genes showing distinct stage- and tissue-specific expression patterns. The scale: averaged log_2_ “relative RPKM value” of all genes in each cluster.

Transcriptional responses to auxin are continuously evaluated and result from the interplay of a number of elements that conform to the signaling pathway (Weijers and Wagner, 2016). We restricted our analysis to the receptacle as the main focus of our research. The pathway starts with the auxin receptor genes *TIR1* and *AFB* (Dharmasiri et al., 2005). In the *F. vesca* genome, four auxin receptors are present, while three are expressed in *F. ananassa* (Supplementary Figure S7). Their study in the receptacle shows that *FaTIR1* slightly increases its expression from the green to red stage, while the expression of *FaAFB2* decreases, and *FaAFB5* remains constant (**Figure 6A**). The analysis was extended to the repressors *Aux/IAA* and the transcription factors *ARFs*, whose interaction is critical to trigger the transcriptional response to auxin. In most species, a high number of members have been found for these gene families. Thus, in *F. vesca*, 21 Aux/IAA and 19 *ARF* genes have been identified (Kang et al., 2013). The possible selectivity of the Aux/IAA-ARF interactions determined by their structural features (Piya et al., 2014) and their co-expression pattern would account for the specificity of the auxin involvement in various developmental processes (Weijers and Wagner, 2016). Therefore, the expression in the developing receptacle of the corresponding genes of *F. ananassa* (*FaAux/IAA* and *FaARF*) was analyzed in the RNA-seq expression data. A total of 19 *FaAux/IAA* genes were expressed in ripening receptacle of strawberry fruit with different patterns (**Figure 6B**). Most of them showed the highest expression at the green stage and then continuously decreased up to the red stage, in agreement with the occurrence of auxin in the green receptacle (**Figure 6B**). However, three genes showed a different pattern. The *FaAux/IAA8a* gene showed the highest expression at all stages, with a small increase from the green to red stage. Two other genes*, FaAux/IAA14b* and *FaAux/IAA11*, increased their expression up to a maximum at the turning stage and then decreased (**Figure 6B**). The expression of the *FaARF* genes is shown in **Figure 6C**. Most of the genes showed decreasing expression, with minor changes, from the green to red stage, but the *FaARF6a* gene, whose expression was the highest in the receptacle compared to the other *FaARFs*, showed a dramatic increase from 77 FPKM at the green to 236 FPKM at the red stage (**Figure 6C**).

Clustering by co-expression analysis of the *FaAux/IAA* and *FaARF* genes in the receptacle during ripening was performed to identify the possible positive interactions between members of the two gene families (**Figure 6D**). Two main clusters are formed. One of them (upper) includes those genes with the highest decrease in the transition from the green to red stage. The other cluster includes genes that showed variable and not very drastic changes in expression during the growth and ripening of the receptacle. In the lower part of this cluster are grouped, in a sub-cluster, those genes whose expression increased with the progression of ripening (*FaAux/IAA11, FaARF6a*, *FaAux/IAA14b*, *FaAux/IAA33*, and *FaARF16c*). In this group, the two genes showing the highest expression level and highest change in the transition from the green to red stage are *FaAux/IAA11* and *FaARF6a.*

**FIGURE 7:**
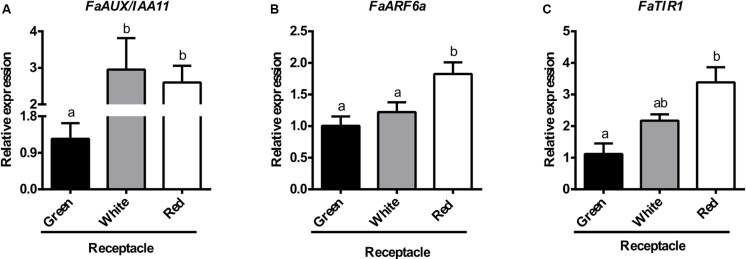
Relative transcript levels of *FaAUX/IAA11* **(A)**, *FaARF6a* **(B)**, and *FaTIR* **(C)**, as determined by qRT-PCR in different developmental stages of receptacle. Bars represent the mean of three independent biological samples ± SE. Different letters indicate a significant difference between samples according to the corresponding ANOVA (*P* < 0.05).

The values obtained for the expression of key genes of the auxin reception and signaling pathway in the ripening receptacle by RNA-seq were validated by qRT-PCR. The results, shown in **Figure 7**, confirm the RNA-seq data, i.e., there is an increase in the expression of the genes *FaAux/IAA11* (**Figure 7A**) and *FaARF6a*, (**Figure 7B**), jointly with the auxin receptor *FaTIR1* (**Figure 7C**), parallel to the ripening process. Altogether, these results point to the operation of the auxin responsive components of the strawberry receptacle at the ripe stage.

In relation to a proteasome-independent auxin signaling pathway, a gene with high homology to the Arabidopsis auxin binding protein *ABP1* (Sauer and Kleine-Vehn, 2011) was identified in *F. vesca*, being its expression in *F. ananassa* higher in achene than in receptacle, where did not show significant differences along ripening (Supplementary Table S2).

### Transient Silencing of *FaTAR2* in Ripening Receptacle Alters the Auxin Response

**FIGURE 8:**
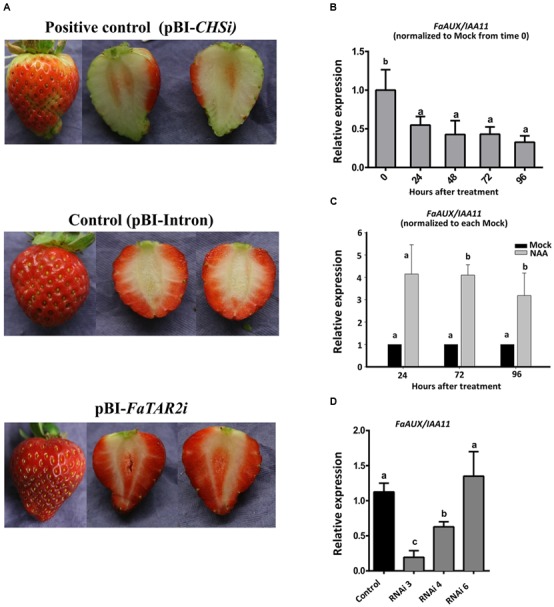
**(A)** Strawberry transgenic fruits agroinfiltrated with empty vector (control), with the FaTAR2-RNAi construct (RNAi) and with *Chalcona synthase*-RNAi, FaCHS-RNAi, as a positive control at 7 days after injection. **(B)** *FaAUX/IAA11* expression by qRT-PCR of de-achened green strawberry fruits and covered with a lanoline paste. **(C)** Time course expression of *FaAUX/IAA11*, by qRT-PCR, of the receptacle of strawberry fruits injected, at the turning stage, with a water solution of NAA and the corresponding control (mock solution). **(D)** *FaAUX/IAA11* expression in silenced fruit agroinfiltrated with empty vector (control) and with FaTAR2-RNAi construct. Error bars indicate +SE of three biological replicates. Different letters indicate a significant difference between samples according to the corresponding ANOVA (*P* < 0.05).

Not only were the auxin reception and signaling genes expressed in ripe receptacle but so were the genes encoding for Trp aminotransferase (*FaTAA1*, *FaTAR2*) (Supplementary Figure S3). This result points to the synthesis of this hormone within this organ at this stage. Therefore, it was possible to interfere with the auxin biosynthetic pathway in this organ at this stage. In strawberry, transient silencing in fruits is a valid alternative to permanent transformation for those genes expressed in this organ (Hoffmann et al., 2006). Thus, RNAi silencing of the biosynthetic gene with the highest value of expression in the red receptacle, *FaTAR2*, was performed. Two of six fruits injected with the *RNAi* construct showed a significant reduction in the expression of *FaTAR2* gene in the receptacle (Supplementary Figure S8). Silencing of *chalcone synthase* (*CHS*) was used as the positive control (Hoffmann et al., 2006). The phenotype of the silenced fruits did not show any apparent difference in relation to the control, but the CHS-silenced fruits showed a characteristic lack of color development (**Figure 8A**).

The silencing of *FaTAR2* is expected to be accompanied by a decrease in the auxin content. The small amount of the sample made it unfeasible to quantify the hormone in the silenced fruits. However, transcript detection of auxin-regulated genes was a valid alternative. The *Aux/IAA* genes are transcriptionally up-regulated by auxin treatment, likely as part of a feedback control mechanism (Abel et al., 1994; Krogan and Berleth, 2015). Thus, we tested whether the *FaAux/IAA11* gene, which is highly expressed in the ripe strawberry receptacle (**Figure 6B**), was sensitive to auxin changes. For this purpose, achenes were removed from green fruits and the expression of *FaAux/IAA11* followed in the receptacle. The expression of this gene clearly decreased after 24 h removal of the achenes, and continued up to 96 h (**Figure 8B**). Another set of fruits, at the turning stage, were injected with a solution of NAA. The expression of *FaAux/IAA11* in the receptacle was increased in comparison with fruits injected with the mock (**Figure 8C**). These results confirmed the effect of auxin on the transcription of this gene. Thus, the decreased expression of *FaAux/IAA11* in the *FaTAR2*-silenced receptacle (**Figure 8D**) might be an indication of a reduced auxin concentration in this organ in comparison with control fruits and fruits where silencing was not effective. Altogether, these results support the synthesis of auxin in the red receptacle. This process is accompanied by a high expression of genes of the auxin reception and signaling pathway, such as *FaTIR1*, *FaAux/IAA11*, and *FaARF6a*, in this organ at this stage.

## Discussion

### Auxin Participates in Cell Processes Taking Place in the Ripening Receptacle

During recent years, there has been a continuous advancement in the comprehension of its synthesis, transport, perception, and signaling (Ljung, 2013; Adamowski and Friml, 2015; Kramer and Ackelsberg, 2015; Salehin et al., 2015; Weijers and Wagner, 2016). The study of these components in different organ/cell scenarios has been useful to reveal their involvement in cellular processes such as cell division, cell enlargement (Velasquez et al., 2016), cell differentiation (Yang and Wang, 2016), and subcellular processes such as remodeling of the cell wall (Pacheco-Villalobos et al., 2016) and lignin biosynthesis (Zhang et al., 2014). Most of them occur at some stage of strawberry fruit development.

The global transcriptional data in the two parts of the strawberry fruit, the achene and the receptacle, separately showed a specific pattern for gene expression of every member of the gene families *FaPIN, FaAUX1/LAX*, *FaTIR1/AFB*, *FaTAA1/TAR*, *FaAux/IAA*, and *FaARF*. Focusing on the receptacle, it is noteworthy the high expression of auxin transport genes at the green stage, and the enhanced transcription of some members of the auxin perception and signaling families at the red stage.

Our expression analysis of the genes encoding tryptophan amino transferases, whose *in vitro* activity of the enzymes has been demonstrated, supports that active auxin biosynthesis occurs in the green achenes and decreases thereafter. The pattern of expression of the auxin transporters supports that active transport is occurring to or from the receptacle. An important observation is the maintenance of auxin content in the receptacle of the ripe fruits, something that was previously reported many years ago but went relatively unnoticed. Analysis of the free auxin content separately in the achene and the receptacle revealed that the levels peaked at the same stage in both organs, corresponding to the transition from free nuclear to cellular endosperm in the achene and the initiation of the exponential growth of the receptacle. Afterward, the auxin content decreased in both tissues, but a late increase was found during ripening in the receptacle (Archbold and Dennis, 1984). The IAA values measured by us in the receptacle are in the range of those previously reported for the receptacle of wild *F. vesca* (Osorio et al., 2011) and for the white receptacle of the cultivated strawberry (Symons et al., 2012). They are approximately 10-fold lower than the values in other plant tissues, such as Arabidopsis roots (Basu et al., 2011) and tomato roots, leaves and fruits (Albacete et al., 2008; Pattison and Catalá, 2011), and more than 20-fold lower than in the achenes at the white stage (Symons et al., 2012). Thus, even at a low level, auxin is present in the ripe receptacle, and this is a novelty of the present work. Moreover, the increase in the expression of *FaTAR2* with ripening, as well as in specific members of the *FaAux/IAA* (*FaAux/IAA11*, *FaAux/IAA14b*, *FaAux/IAA33*) and *FaARF (FaARF6a, FaARF16c)* families, points to a cell-autonomous auxin synthesis and action in the ripe receptacle.

The strawberry receptacle consists of a fleshy pith at the center surrounded by cortical tissue containing parenchymal and epidermal cells. Vascular bundles traverse the pith and the cortex to the achenes (Perkins-Veazie, 1995). During growth and ripening, the different cell types differ in their division rate, final cell size, cell wall composition and metabolic activity (Darrow, 1966; Fait et al., 2008; Nasopoulou et al., 2014). The processes associated with these changes involve cell wall remodeling, lignin biosynthesis and drastic changes in primary and secondary metabolism. Therefore, we hypothesize that the action of auxin in the ripening receptacle might be spatially limited to specific cell types, and associated with some of these processes.

### Specific Members of the Reception and Signaling Machinery Are Involved

The high number of Aux/IAA and ARF proteins in most of the species offers a wide range of combinatorial interactions that would account for the numerous specific plant processes in which auxin participates. This complex interaction network translates the local accumulation of auxin to gene expression by specific ARF transcription factors (Weijers and Wagner, 2016). In the sequenced genome of diploid *F. vesca*, 21 members of the *Aux/IAA* family and 19 members of the *ARF* family have been identified (Kang et al., 2013). Our analysis of the *F. ananassa* transcriptome in this species showed the expression of 19 *Aux/IAA* and 16 *ARF* members of these families in fruit, at different developmental stages, leaf and root. Their diverse expression pattern reveals the existence of a complex auxin signaling machinery that is prepared for the action of this hormone in different processes. Moreover, co-expression analysis might identify the possible Aux/IAA-ARF interacting partners involved in specific tissue/stage processes. Thus, in the ripening receptacle, where we here report the synthesis of auxin by an active FaTAR2 enzyme, the *FaAux/IAA11* and *FaARF6a* clustered together. The nomenclature of the *F. vesca* genes of these families (Kang et al., 2013), as well as the *F. ananassa* genes, was determined by their sequence closeness to the Arabidopsis genes. In this species, it was found that AtARF6 interacted in a yeast two-hybrid assay, confirmed by bimolecular fluorescence complementation, with a number of Aux/IAA proteins, including AtAux/IAA11 (Piya et al., 2014). Moreover, co-expression analysis of the two gene families, *Aux/IAA* and *ARF*, showed that positive correlation in this species was high for *AtAux/IAA11* and *AtARF6* in flower buds and flowers (Piya et al., 2014). Altogether, our results point to FaARF6a as a main final transcriptional regulator, likely interacting with FaAux/IAA11, translating the activity of auxin in the ripening receptacle.

Sequence analysis of *FaARF6a* identified in the encoded protein the characteristic features of the class A ARFs (Supplementary Figure S9), which includes the N-terminal B3 DNA-binding domain, flanked by the dimerization domains, the Q-rich activation domain, and the C-terminal PB1 domain for oligomerization and Aux/IAA-ARF heterodimerization (Guilfoyle and Hagen, 2012; Weijers and Wagner, 2016). The class A ARFs are classified as transcriptional activators (Ulmasov et al., 1999). Thus, it is expected that FaARF6a targets genes with enhanced expression in the receptacle as ripening advances in this organ.

In Arabidopsis, AtARF6 has been demonstrated to play a central role in auxin’s regulation of cell elongation in the hypocotyl (Oh et al., 2014). The identification of AtARF6 targets was performed by chromatin-immunoprecipitation followed by sequencing (ChIP-Seq) analysis and further comparison with genes previously described as auxin-activated (Tiwari et al., 2003). A total of 255 AtARF6 targets were identified, many of them involved in cell elongation. Interestingly, in the ripening receptacle, cell enlargement is a process that increases rapidly when cell division ceases (Cheng and Breen, 1992). At the transition from the green to white stage, the highest increase in the expression of *FaARF6a* occurs. Moreover, in elongating hypocotyl, the DNA binding capacity of AtARF6 was blocked by the gibberellin-inactivated repressor RGA (Oh et al., 2014), which constitutes a good example of gibberellin involvement in the auxin response. In the ripening receptacle, we have previously reported the involvement of gibberellin in its development, with a peak at the white stage, and *FaRGA* expression dramatically decreasing from the green to white stage (Csukasi et al., 2011). Whether *FaARF6a* constitutes a link between auxin and gibberellin in the cell enlargement taking place in the ripening receptacle deserves to be investigated. Interestingly, in strawberry fruits where the *FaGAMYB* gene was silenced, the expression of *FaARF6* was significantly down-regulated (Vallarino et al., 2015). *FaGAMYB* is a GA-responsive transcription factor that plays a key role in the changes occurring in receptacle development prior to its ripening. An analysis of the *FaARF6a* targets during this process would provide information on the specific molecular processes regulated by this gene in the strawberry receptacle. Moreover, cell elongation is not a uniform process in strawberry receptacle development. Thus, there is a distinct gradient in cell size in the central pith and in the cortex, with the smaller cells near the periphery and the larger ones toward the inside (Havis, 1943). In addition, the cortex develops more rapidly than the pith and at a higher relative rate. All of these points mean that the cell elongation in the developing receptacle might be circumscribed to specific groups of cells, depending on time and space. This result must be considered when investigating the molecular events associated with cell elongation and the involvement of specific gene products such as FaARF6a.

## Author Contributions

VV conceived the project. NM-E, MB, SO, and VV planned, designed and supervised the research. EE-J, FC, CP, JV, YK, AV, JB, JS-S, and SO performed the experiments. All authors contribute to data analysis and the writing of the manuscript.

## Conflict of Interest Statement

The authors declare that the research was conducted in the absence of any commercial or financial relationships that could be construed as a potential conflict of interest.

## Footnotes

1 http://www.phenomenex.com

2 http://www.megasoftware.net/

3 https://phytozome.jgi.doe.gov/pz/portal.html

4 http://www.rosaceae.org

5 http://www.ebi.ac.uk/Tools/msa/clustalo
