# Corrigendum: Transcriptomic Analysis in Strawberry Fruits Reveals Active Auxin Biosynthesis and Signaling in the Ripe Receptacle

**DOI:** 10.3389/fpls.2017.01305

**Published:** 2017-07-24

**Authors:** Elizabeth Estrada-Johnson, Fabiana Csukasi, Carmen M. Pizarro, José G. Vallarino, Yulia Kiryakova, Amalia Vioque, Catharina Merchante, Javier Brumos, Nieves Medina-Escobar, Miguel A. Botella, José M. Alonso, Alisdair R. Fernie, José F. Sánchez-Sevilla, Sonia Osorio, Victoriano Valpuesta

**Affiliations:** ^1^Departamento de Biología Molecular y Bioquímica, Instituto de Hortofruticultura Subtropical y Mediterranea, Universidad de Málaga-Consejo Superior de Investigaciones Científicas Málaga, Spain; ^2^Dipartimento di Scienze, Università degli Studi della Basilicata Potenza, Italy; ^3^Department of Plant and Microbial Biology, North Carolina State University Raleigh, NC, United States; ^4^Max-Planck Institute of Molecular Plant Physiology Postdam-Golm, Germany; ^5^Instituto Andaluz de Investigación y Formación Agraria y Pesquera, IFAPA-Centro de Churriana Málaga, Spain

**Keywords:** auxin, fruit, strawberry, transcriptome regulation, ripening

Catharina Merchante was not included as an author in the published article. The authors apologize for this error and state that this does not change the scientific conclusions of the article in any way.

## Conflict of interest statement

The authors declare that the research was conducted in the absence of any commercial or financial relationships that could be construed as a potential conflict of interest.

