# Supplementary material for: Transcriptomic Analysis in Strawberry Fruits Reveals Active Auxin Biosynthesis and Signaling in the Ripe Receptacle
Source: Front Plant Sci. 2017 May 29;8:889. doi: 10.3389/fpls.2017.00889 (PMC5447041; doi:10.3389/fpls.2017.00889)
Supplement: Supplementary file 1 [file Table_1.docx]

Table S1. Summary of the RNA-Seq study.

**Tissue/sample** corresponds to the different tissues and stages used in the analysis, and the total global results. **Average number of reads** corresponds to the average number (in millions, M) of processed reads of the three replications pools for each sample. **Overall alignment rate** corresponds to the average number of the readsb(as percentage) mapped over the *Fragaria vesca* Whole Genome v2.0.a1 Assembly (http://www.rosacea.org) . **Number of XLOCs FPKM > 0.3** corresponds to the number of putative *loc*i with a number of fragments per kilobase of exon per million of fragments mapped higher than 0.3.

| Tissue/Sample | Average number of reads | Over all alignment rate | Number of XLOCs  FPKM > 0.3 |
| --- | --- | --- | --- |
| Green Achene | 26.8 M | 79.32% | 19,250 |
| White Achene | 29.0 M | 76.06% | 17,994 |
| Turning Achene | 26.4 M | 75.15% | 17,947 |
| Red Achene | 30.3 M | 75.06% | 17,847 |
| Green Receptacle | 33.9 M | 78.87% | 17,918 |
| White Receptacle | 40.7 M | 78.56% | 17,353 |
| Turning receptacle | 31.6 M | 78.93% | 16,521 |
| Red receptacle | 37.6 M | 78.62% | 16,509 |
| Leaf | 31.9 M | 78.96 | 19,045 |
| Root | 30.8 M | 75.79 | 19,146 |
| Total |  | 77.53 | 23,702 |
