# Supplementary material for: Transcriptomic Analysis in Strawberry Fruits Reveals Active Auxin Biosynthesis and Signaling in the Ripe Receptacle
Source: Front Plant Sci. 2017 May 29;8:889. doi: 10.3389/fpls.2017.00889 (PMC5447041; doi:10.3389/fpls.2017.00889)
Supplement: Supplementary file 3 [file Presentation_1.PDF]

Supplemetary Material

Transcriptomic analysis of auxin synthesis and signalling genes in developing receptacle of strawberry fruit

Elizabeth Estrada[1#] , Fabiana Csukasi[1#], Carmen Martín Pizarro[1], José G. Vallarino[1], Yulia Kiryakova[2], Amalia Vioque[1], Javier Brumos[3], Nieves Medina-Escobar[1], Miguel A. Botella[1], José M. Alonso[3], Alisdair R. Fernie[4], José F. Sánchez-Sevilla[5], Sonia Osorio[1,*], Victoriano Valpuesta[1,*]

Correspondence: Victoriano Valpuesta and Sonia Osorio (valpuesta@uma.es; sosorio@uma.es)

Supplementary Figures:

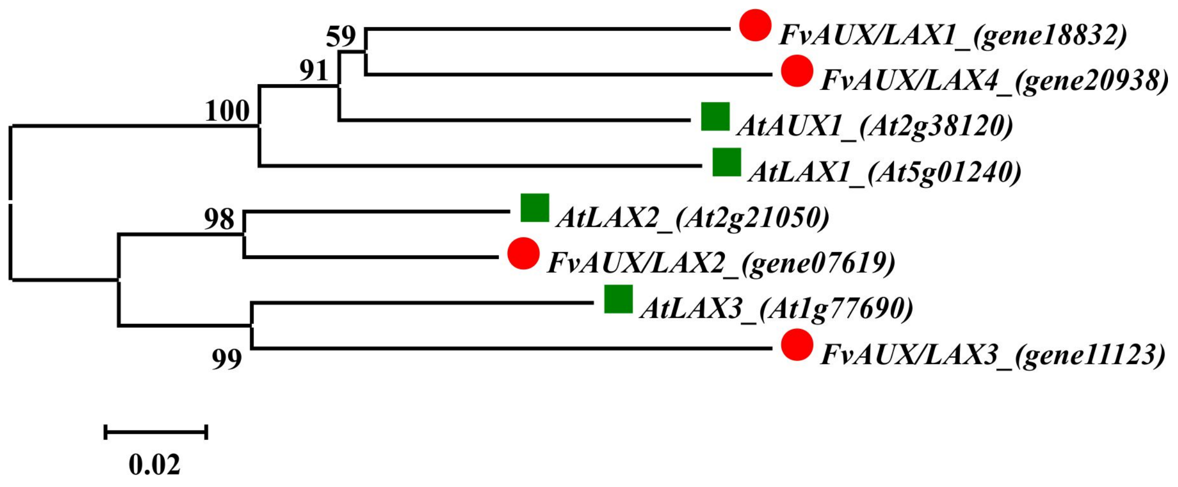

Supplementary Figure 1. Dendrogram of the alignment for *AUX/LAX* genes from Arabidopsis and strawberry.

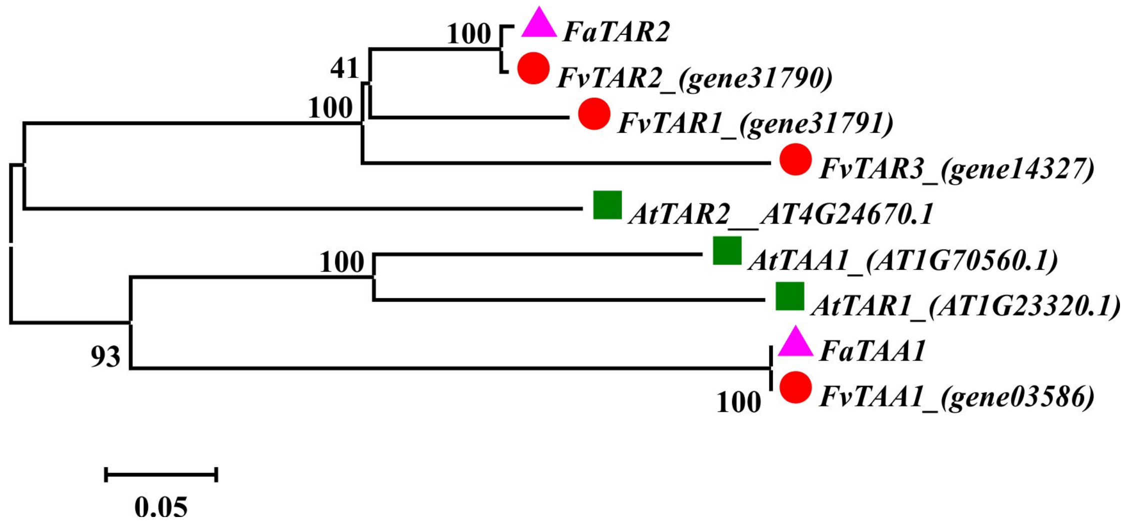

Supplementary Figure 2. Dendrogram of the alignment for *TAA* and *TAR* genes from Arabidopsis and strawberry.

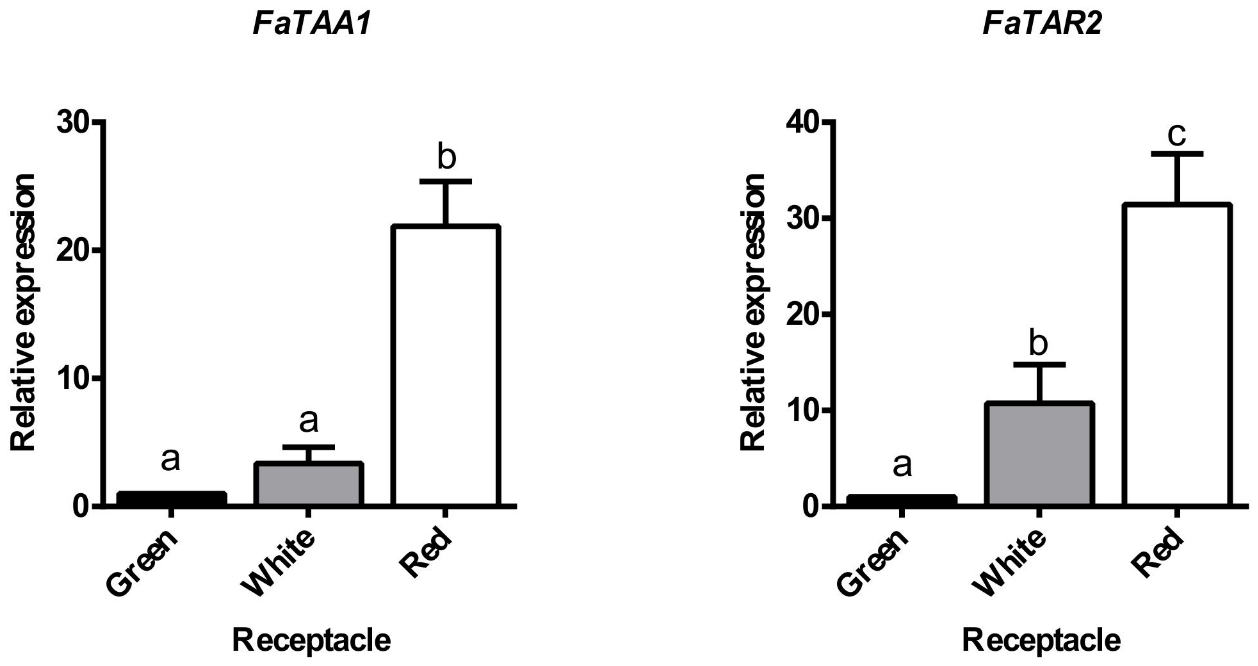

Supplementary Figure 3. Relative expression by qRT-PCR of *FaTAA1* and *FaTAR2* at three developmental stages. Error bars indicate ± SE of three biological replicates . Different letters indicate a significant difference between samples according to the corresponding ANOVA ($P < 0.05$).

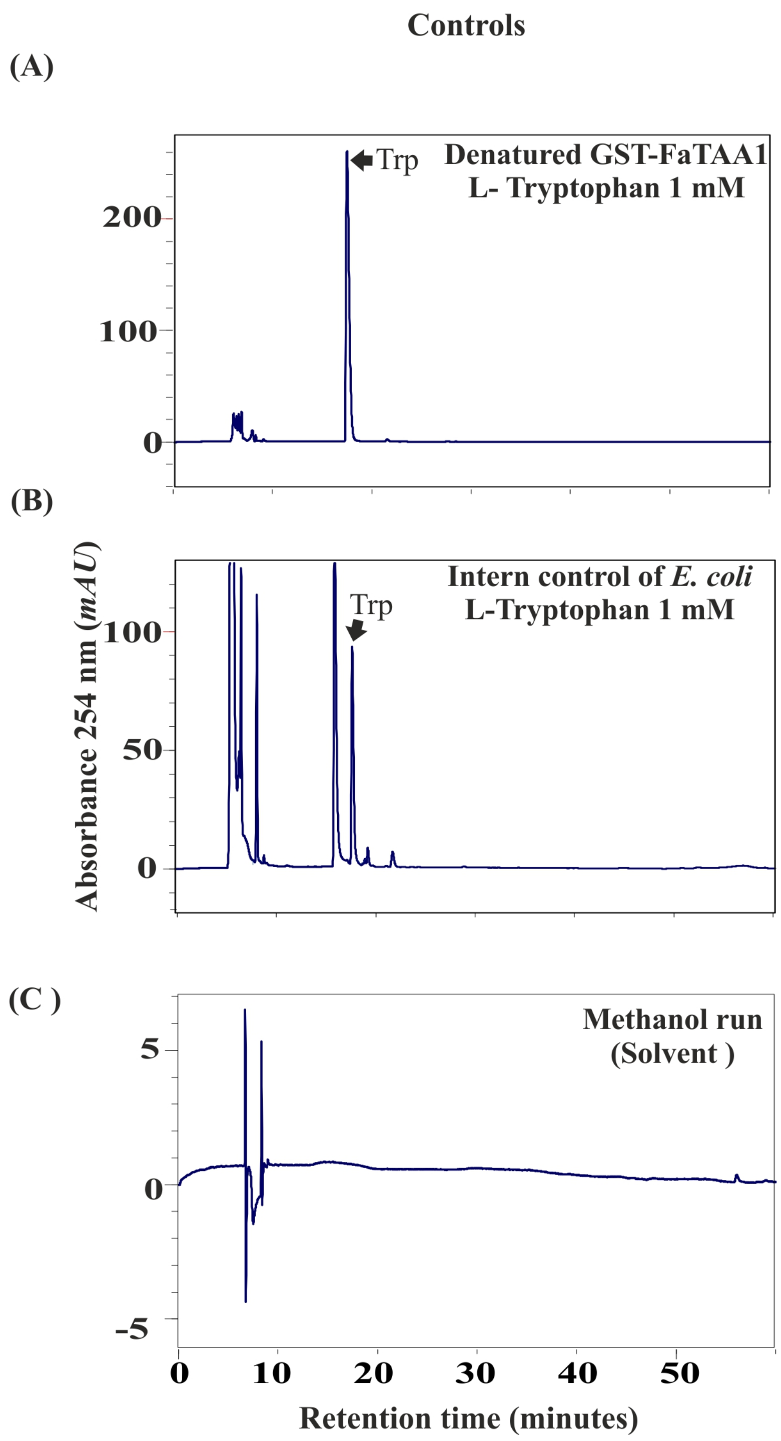

Supplementary Figure 4. Trp aminotransferase (AT) activity of FaTAA1. HPLC chromatograms of the products of the in vitro AT reactions catalyzed by the purified and boiled GST-FaTAA1 protein (negative control).

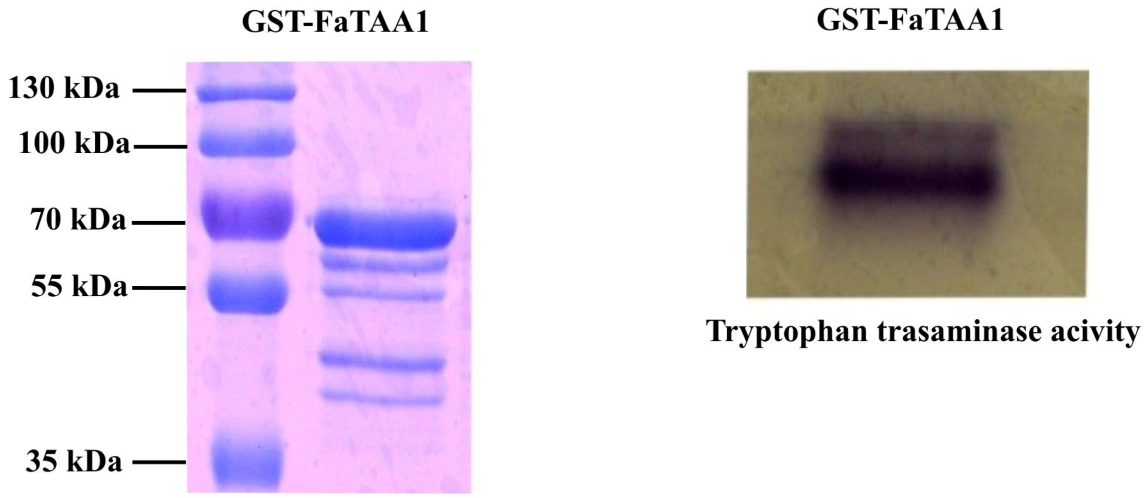

Supplementary Figure 5. Trp aminotransferase (AT) activity of FaTAA1. Purified recombinant GST-FaTAA1 shows AT in an in-gel assay using Trp as a substrate.

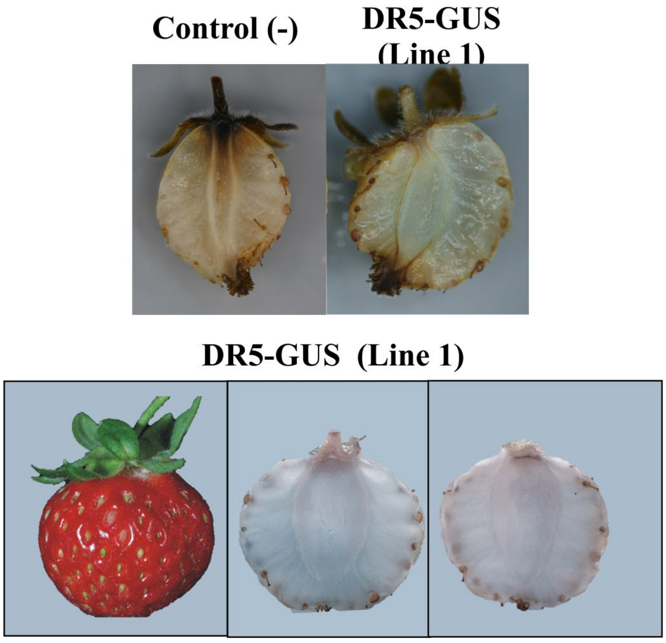

Supplementary Figure 6. DR5-GUS expression pattern in red fruit.

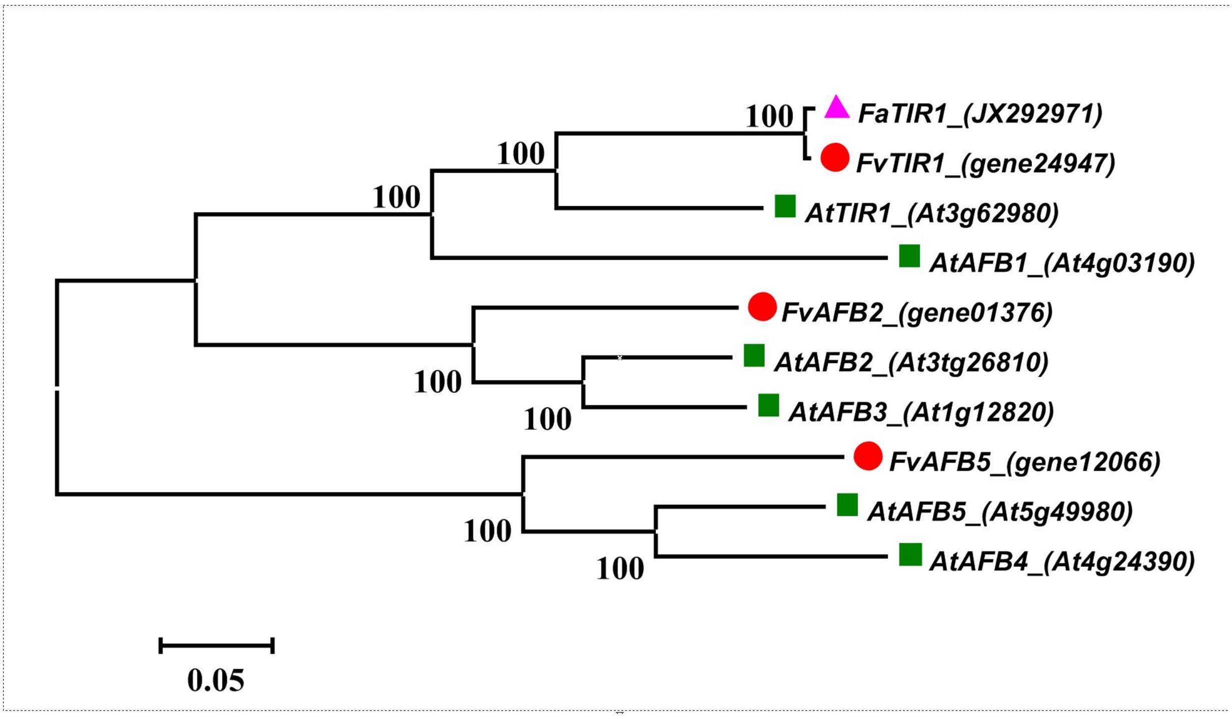

Supplementary Figure 7. Dendrogram of the alignment for *TIR1* and *AFB* genes from Arabidopsis and strawberry.

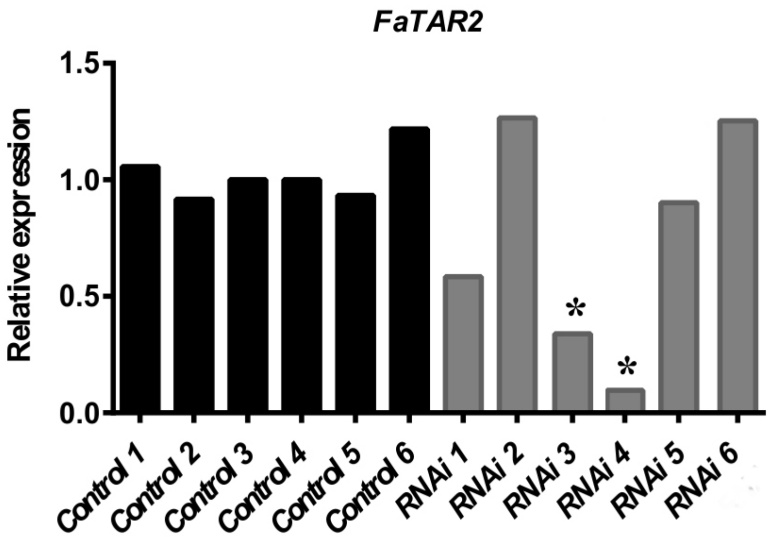

Supplementary Figure 8. Expression of *FaTAR2* by quantitative real-time PCR (qRT-PCR) in different strawberry fruits agroinfiltrated with empty vector (control) and with the FaTAR2-RNAi construct (RNAi).

Supplementary Figure 9. Nucleotide sequence, and amino acid translation of the ORF, corresponding to the transcript of the *FaARF6a* gene assembled from the RNAseq data using the Trinity software. The more abundant nucleotide in the polymorphic positions are shown. Known domains are indicated in the Table, and they are marked as underlined in the amino acid sequence, with the key residues in bold.

DOMAIN	Code	DESCRIPTION	Amino acid Position	E-value of BLASTX
**B3_DNA**	**cd10017**	**Plant-specific B3-DNA binding domain**	**126-227**	**2.40e^{-21}**
**Auxin_resp**	**pfam06507**	**Auxin response factor**	**258-336**	**2.33e^{-39}**
**AUX_IAA**	**cl03528**	**AUX/IAA family**	**745-838**	**2.54e^{-23}**

LOC101306795 auxin response factor 6 XM_004293453.2 XP_004293501.1

1 agagagagagagagagagagagagagagagagagagtgtgtgtgtaaagcaatagtggcaaa
61 tccaccaagcaagcagtagagtggtactagactagactctactaagaagtaagaacacaa
121 agaacacggacacaccaccaccgctaccagacacatcaagcaaattagctttctgtttcc
181 ataaccggaagactttggatcaaaacccaactcttggttttctatatttgcgcatgttct
241 agttcaaaacagtatgtgtattggtctgtgctaggctttgttcatcacttagctttggtt
301 tttctttggctcttagataataatgtgtaggctgatgaaggtttcatttgggtttttatg
361 gtgtgagatctggtgctttaaggtggcataggctttgggtgttctgaggctgagatctgt
421 tgcttctttcagcagtgttgggtttttaggctattggggttttctggggtttgctgtaaa
481 aagcttggtttttgagattggttatttgggttgagtaaagttttgatttttt**t**gaagctgt
541 gagtcttgaggctcagttccagaggtgaaggtagggtgtttcaaatgttgaggcttttc
601 attgaaggttggggaatgaggtgtttaattagaggttggacattctgggggtggggctg
661 aggttgatcaggtgcgaatgaggctgaagttgattgtgttttgtagtgaaaccggtttcg
721 gttgaaaatcagaagttgggttctttatggtatgctttgcatgaatgtgaag**c**ttcctgc
781 ttttgtaaagttgtgcatcaagtttttttttccttttctgggatttgaatggggttttgtc
841 cggtggagatatgagattgtgtagtgaaaagaaaggaagtgaagaaaaggattgttctgt
901 ggtttttaggggattatttgtgtacactgttcagttattgtgatttaagtactttttgatt

1 M R L S S A G F S P Q P Q E G E K
961 cactgcaagATGAGGCTCTCTTCTGCTGGCTTTAGTCCTCAACCCCAAGAAGGGGAAAAG

18 R V L N S E L W H A C A G P L V C L P A
1021 AGAGTTTTGAATTCTGAACTTTGGCATGCTTGTGCCGGTCCTCTTGTTTGTCTACCTGCT

38 V G S R V V Y F P Q G H S E Q V T A S T
1081 GTTGGTAGCCGTGTAGTTTATTTTCCTCAAGGTCACAGTGAACAGGTTACTGCTTCAACC

58 N M E V D S H I P N H P S L P P Q L I C
1141 AACATGGAAGTGGATTCCCATATTCCTAATCACCCAAGCTTACCTCCACAACTTATCTGT

78 Q L H N V T M H A D V E T D E V Y A Q M
1201 CAACTACACAATGTGACGATGCATGCAGATGTTGAGACAGATGAAGTATATGCACAGATG

98 T L Q P L N P Q E Q K D G Y L P A G L G
1261 ACCTTGCAACCATTGAATCCACAAGAGCAAAAGGATGGCTACCTTCCAGCAGGATTAGGC

118 S P N K Q P T N Y **F** C **K** T **L** **T** A **S** **D** T **S**
1321 AGCCCTAACAAACAGCCAACAAACTATTTCTGTAAAACTTTGACAGCCAGTGACACTAGT

138 T H **G** G F S V **P** R R A **A** E K V F **P** **P** L D
1381 ACCCACGGTGGTTTTTCTGTTCCTCGCCGGGCTGCTGAAAAAGTTTTTCCTCCATTGGAC

158 F S Q Q P P A Q **E** L I A R **D** L H D N E **W**
1441 TTCTCCCAACAACCACCAGCGCAAGAGTTAATTGCAAGGGATCTGCATGATAATGAATGG

178 K F R H I F **R** G Q P K **R** H L **L** T T **G** **W** S
1501 AAATTTAGACACATATTTCGCGGCCAGCCTAAAAGGCATCTCCTTACAACAGGGTGGAGT

198 V **F** **V** S **A** K R **L** V A **G** **D** S V L **F** I W N E
1561 GTGTTTGTAAGTGCTAAAAGACTGGTTGCTGGAGACTCGGTTCTTTTCATCTGGAATGAA

218 K **N** Q L L L G **I** R **R** A N R P Q T V M P S
1621 AAAAATCAACTACTCCTTGGTATCCGGCGAGCTAACCGACCACAAACTGTGATGCCTTCA

238 S V L S S D S M H L G L L A A A A H A A
1681 TCAGTTTTATCAAGTGATAGCATGCACTTGGGACTTCTTGCTGCTGCAGCTCATGCAGCT

258 **S** **T** N **S** R **F** T I **F** **Y** **N** **P** **R** **A** **S** P **S** **E** **F** **V**
1741 TCAACAAATAGTCGTTTCACCATATTTTATAATCCAAGGGCTAGCCCATCAGAGTTTGTC

278 I **P** L **A** **K** **Y** I **K** **A** V Y H T H I **S** **V** **G** **M** **R**
1801 ATTCCCCTGGCCAAGTACATTAAGGCAGTCTATCATACTCATATTTCTGTTGGCATGCGT

298 **F** R **M** L **F** **E** **T** **E** E **S** **S** V **R** **R** Y M **G** **T** **I** T
1861 TTTAGGATGTTATTTGAAACAGAGGAATCAAGTGTCCGACGCTACATGGGAACCATAACT

318 **G** I **S** **D** **L** **D** A A **R** **W** **P** **N** **S** H **W** **R** **S** V K V
1921 GGCATAAGTGACCTAGATGCTGCTCGGTGGCCTAATTCACATTGGCGTTCAGTCAAGGTG

338 G W D E S T A G E R Q P R V S L W E I E
1981 GGCTGGGACGAATCCACAGCTGGGGAGAGGCAGCCAAGAGTGTCCCTGTGGGAGATTGAA

358 P L T T F P M Y P S S F P L R L K R P W
2041 CCATTAACAACATTTCCCATGTATCCATCTTCGTTCCCCCTTAGGCTTAAGCGGCCATGG

378 P P G L P S Y N G L R E D D H N M N S P
2101 CCACCTGGACTACCCTCTTATAATGGTTTGAGGGAAGATGACCATAACATGAATTCTCCA

398 L L W L R G D T G D R G I Q S L N Y H G
2161 CTTTTGTGGCTTCGAGGAGATACTGGAGACCGTGGAATCCAGTCTTTGAACTATCATGGC

418 I G V T P W M Q P R F D A S M I G L Q T
2221 ATTGGGGTCACACCATGGATGCAACCCAGGTTTGATGCTTCTATGATTGGCTTGCAGACA

438 D M Y Q A M A A A A L Q E M R G V D P S
2281 GACATGTACCAAGCTATGGCTGCTGCTGCCCTTCAGGAGATGAGGGGCGTAGACCCTTCC

458 K L L P T S L L Q F Q Q T Q N L S S R S
2341 AAATTGCTACCTACGTCCCTTCTGCAGTTTCAGCAAACCCAAAACCTCTCCAGCAGGTCT

478 A A L M Q P Q M V Q E S Q S Q Q A F L Q
2401 GCTGCCTTAATGCAGCCCCAGATGGTGCAAGAGTCTCAATCTCAACAAGCCTTCCTCCAA

498 G V E E I R Q S Y S Q T P T Q S H L Q H
2461 GGTGTTGAAGAAATTCGTCAGTCATATTCTCAGACTCCAACGCAGTCGCACCTGCAGCAT

518 Q L Q H Q N S F S N Q Q Q Q I L D H Q Q
2521 CAATTGCAGCACCAAAACTCATTCAGTAATCAACAGCAGCAGATCCTTGATCACCAACAG

538 I P S A I S S M N Q F A S A S Q S R S P
2581 ATTCCAAGTGCTATCTCTTCCATGAATCAGTTTGCTTCTGCTTCTCAATCCCGGTCACCA

558 S F Q V I T S P C Q Q Q S F P D S N G N
2641 TCTTTTCAAGTTATCACCTCAC**T**ATGCCAACAACAGAGCTTTCCTGATTCGAATGGGAAC

578 S A T S T T L S P L S S L M G S F S Q D
2701 TCTGCAACCAGCACCACATTATCTCCCTTGAGCAGTCTTATGGGTTCATTTTCGCAGGAT

598 E S S N L L N V P R T N P L L S S S G W
2761 GAATCTTCCAACCTGCTCAACGTGCCTAGAACTAATCCTTTACTGTCATCTTCTGGCTGG

618 P S K R A A I E P L L S S G V P Q C V L
2821 CCATCTAAGCGAGCAGCAATTGAACCTCTTCTTTCATCTGGAGTTCCTCAATGTGTTCTG

638 P Q V E Q L G P P Q T T I S H S P I S L
2881 CCCCAGGTGGAACAATTGGGACCTCCCCAAACTACCATCTCTCATAGTCCTATTTCATTA

658 P P F P G R E C S I D Q E G S T D P Q T
2941 CCACCCTTTCCTGGGAGAGAGTGCTCAATAGACCAAGAAGGAAGCACCGATCCTCAAACC

678 H L L F G I N M S N L R A V G S D S V S
3001 CATCTTTTATTTGGTATCAATATGTCAAACCTTAGAGCAGTTGGCAGCGATAGTGTTTCT

698 T T I H F P S N Y M S T T E T D F S L N
3061 ACAACGATACATTTTCCTTCTAATTATATGAGCACTACAGAAACTGATTTTTCTCTCAAT

718 P A V T P S N C I D E S G F L Q S P E N
3121 CCGGCAGTTACGCCTTCTAATTGCATTGACGAATCAGGTTTCCTTCAGTCTCCAGAAAAC

738 V G H E N Q P N **G** N F **V** **K** **V** Y K S **G** S **Y**
3181 GTGGGCCATGAAAACCAACCAAATGGAAATTTTGTTAAGGTCTACAAGTCAGGGTCCTAC

758 G **R** S L **D** I T K F S **S** **Y** H **E** **L** R R E **L** A

3361 TTTGTTGATCGAGAGAATGATGTTCTTCTCCTCGGGGATGACCCCTGGCCGGAGTTTGTA

818 N **S** V W C I K **I** L S P Q **E** V Q Q M G K **R**
3421 AATAGTGTGTGGTGCATCAAAATACTCTCACCACAAGAAGTGCAGCAAATGGGAAAACGA

838 G L E L L K S V P M Q R L S S N S C D D
3481 GGCCTAGAACTTCTGAAGTCTGTCCCAATGCAGAGGCTCTCCAGCAATAGTTGTGATGAT

858 Y G G S R Q D S R N L S S G I T S V G S
3541 TATGGAGGAAGCCGGCAGGACTCAAGAAATTTGAGTAGTGGGATAACTTCGGTGGGATCA

878 L E Y *
3601 CTCGAGTATTGAactacttaaccagttaagatatcattctctcgatcaatactctgtaat
3661 atttgcatctcttttgacccttttgattaagagttggaagggtgcctaatactagcttaa
3721 aaactttagtttatatataagctaatactgtaattaaatgttacttatcttcttttggta
3781 tattcttctgtcagaaaagtacaatacatatatgtattaggagattaatttttgtgaaaa
3841 atagcctcaatatattgcaatctatttctgatgagggtctatacgaa
